# A Simple Inner-Stopper Guarded Trephine for Creation of Uniform Keratectomy Wounds in Rodents

**DOI:** 10.18502/jovr.v16i4.9743

**Published:** 2021-10-25

**Authors:** Peter B. Le, Fang Chen, David Myung

**Affiliations:** ^1^Department of Ophthalmology, Stanford University School of Medicine, CA, USA; ^2^VA Palo Alto Health Care System, Palo Alto, CA, USA; ^3^Department of Chemical Engineering, Stanford University, CA, USA

**Keywords:** Anterior Lamellar Keratoplasty (ALK), Corneal Defect Model, Inner-stopper Guarded Trephine, Keratectomy, Rat Corneal Wound Model, Trephine Design

## Abstract

**Purpose:**

Creating controllable, reproducible keratectomy wounds in rodent corneas can be a challenge due to their small size, thickness, and the lack of usual tools available for human eyes such as a vacuum trephine. The purpose of this work is to provide a consistent, reproducible corneal stromal defect in rats using a simple, economical, and customized inner-stopper guarded trephine.

**Methods:**

The inner-stopper guarded trephine is used to induce a circular wound in rat corneas. After trephination, the corneal flap can be removed by manual dissection using a blunt spatula. We used optical coherence topography (OCT) to measure the defect wound depth induced in *ex vivo* rat eyes.

**Results:**

Despite a minor learning curve, this simple device enables depth control, reduces variability of manual keratectomy wound depth in rats, and decreases the risk for corneal perforation during keratectomy. Corneal defect creation was highly reproducible across different researchers and was independent of their surgical training.

**Conclusion:**

This inner-stopper guarded trephine can be utilized and applied to pre-clinical testing of a wide range of corneal wound healing therapies, including but not limited to biotherapeutics, corneal prosthetics, and regenerative technologies.

##  INTRODUCTION

The cornea is the transparent, outermost part of the eye that is responsible for protecting intraocular eye structures and focusing light onto the retina.^[1]^ It is susceptible to exposure-related injuries such as burns involving chemical, thermal, and radiation sources, as well as physical trauma.^[2]^ Diseases and injuries to the cornea result in impaired vision due to scarring, clouding, thinning, among others, and in advanced cases, blindness.^[3]^ Many ongoing therapeutic efforts in corneal research include the use of pre-clinical animal models on rodents such as rats.^[4,5]^ This disease model can be used to investigate potential corneal wound healing treatments, such as cell transplantation, amniotic membranes, fibrin gel, as well as polymers and biopolymers.^[6]^ Larger animal models with similar ocular anatomy to humans have been widely used to study wound healing in the cornea. We recently developed a 3D printable modified trephine that can create consistent anterior lamellar keratectomy wounds in rabbits.^[7]^ This trephine was used to create defects to study the ocular wound-healing effects of novel *in situ* forming hydrogels in rabbits.^[8,9,10]^ The development of a similar trephine for rats is equally important because one of the many advantages of using rodents is the availability of visual function tests available for rodents, but not larger animals.^[11,12]^ Also, the cost for rat studies are much lower than that of larger animals. Therefore, rats serve as an important model for the study of wound healing in the cornea. The size of their cornea is large enough to allow for the study of wounds and subsequent tissue collection and analyses.^[13]^ However, rats have smaller eyes and their average central corneal thickness (CCT) is 159 μm,^[14]^ so studying cornea wound healing in rats involves producing defects with precision and reproducibility. Thus, there is a need to develop a reliable system to create consistent and controlled corneal defects in rats in a way that minimizes the risk of corneal perforation. Here, we report an inner-stopper guarded trephine to create consistent anterior lamellar keratectomy in rats.

There are many variables to consider when deciding on a wound model for studying the cornea. Some studies require only debridement of the epithelium, thereby preserving the basement membrane, while others require wounds that penetrate the basement membrane and extend into the stroma.^[15,16,17]^ The proposed method will focus on the latter because it allows the researcher to consistently remove corneal stromal tissue for studies involving wounds that penetrate the stroma. Currently, there are several techniques available to produce anterior wounds to the corneal stroma. Manual keratectomy (MK) involves applying and rotating a sharp trephine or circular biopsy punch to the surface of the cornea, gradually increasing pressure to produce a wound and subsequently excising the corneal flap. Another method, photorefractive keratectomy (PRK), uses an excimer laser to ablate corneal tissue. Compared to MK, laser excimer ablation is more precise and reduces the variability in the depth of the injury.^[18]^ However, laser ablation requires extensive training by the surgeon and has high equipment costs.^[19,20,21]^ Alternatively, microkeratomes are another tool that can be potentially utilized to create a corneal stromal defect.^[22]^ However, there are no microkeratomes that currently exist for rats. Therefore, a method for creating corneal wounds using manual, mechanical tools would be an important advance for preclinical research on small animals.

Of the reported studies involving the need to model corneal injuries in rats, there is no systematic model to produce consistent manual corneal defects. Achieving similar corneal cut depths across eyes is difficult to achieve manually. To reliably assay the effects of therapeutics on corneal wound healing, confounding variables such as the degree of corneal deformation during trephination must be taken into consideration.

Perforation of the cornea results in leakage of anterior chamber fluid, in which case the globe and consequently the animal may have to be euthanized unless the eye is still usable for study after globe repair with sutures.^[23]^ Accordingly, a corneal defect that is too superficial and does not cut deeply enough will likely be repaired by the animal's innate wound healing mechanisms rather than the treatment itself.^[24]^ Therefore, the purpose of this method is to design a disease model for a consistently deep but non-perforating stromal defect in rat corneas.

We developed a handheld trephine with an inner stopper that allows for reproducible corneal stromal defects in rat eyes. After the trephination, we performed keratectomies on *ex vivo* rat corneas with a blunt spatula and imaged them using optical coherence topography (OCT). We quantified the depth of the excised rat cornea using OCT stromal thickness measurements and demonstrated that the device and protocol can be used to produce defects of consistent depth in rats between different users.

##  METHODS

No live rats were operated on for the data collection that supports the use of this method. However, if the techniques described in this paper are to be used *in vivo*, then researchers should abide with the ARVO statement for the Use of Animals in Ophthalmic and Vision Research.

Rat eyeballs were purchased from Vision Tech (Sunnyvale, Texas, USA) and Pel-Freeze Biologicals (Rogers, AR, USA). 2 mm biopsy punches (RBP-20) were purchased from Robbins Instruments, Inc. (Sunnyvale, CA, USA). Ring forceps (11106-09), micro spatula (10089-11), and fine-point surgical forceps (11445-12) were purchased from Fine Science Tools (Foster City, CA, USA). Teflon polytetrafluoroethylene (PTFE) sheets, 0.127 mm (8569K16) and 2.38 mm (8545K14), were purchased from McMaster-Carr (Elmhurst, IL, USA)

### Creating the Inner-stopper Guarded Trephine

Use a 2 mm biopsy punch to punch a piece of 2.38 mm thick Teflon sheet. This piece of Teflon sheet is used as the inner stopper. Use the same 2 mm biopsy punch in the previous step to punch a piece of Teflon sheet that is 127 µm thick. This thinner piece of Teflon sheet is used as the trephination depth calibrator. Note that this second piece of Teflon can be adjusted to a thickness equal to the desired depth of the corneal defect. Transfer and insert the two Teflon sheets into a new 2 mm biopsy with the inner stopper sheet further away from the blade and the calibrator sheet closer to the blade. Refrain from denting the new 2 mm biopsy punch to keep the blade sharp. Using a blunt needle, push the Teflon sheets from the posterior end of the trephine until two sheets are contacting closely and the calibrator sheet is flush with the edge of the blade. Apply a few drops of cyanoacrylate to the posterior end of the trephine to keep the stopper sheet in place. Remove the calibrator sheet from the anterior of trephine.

### Performing the Keratectomy 

Using the inner-stopper guarded trephine, begin by preparing the *ex vivo *rat eyeball or animal for aseptic surgery. Place the inner-stopper guarded trephine on the center of the rat cornea. Press the trephine blade toward the cornea. A dent will form on the cornea without cutting into the corneal stromal tissue. Rotate the trephine in one direction (either clockwise or counter-clockwise) for at least 90°, and then rotate again in the other direction for 90°. Repeating the rotation multiple times will allow the blade cut into corneal stroma. Discontinue rotation when the trephine blade does not go into the stroma any deeper. Gently pull out the trephine from the eye.

### Removing the Stromal Tissue

To remove the layer of anterior stroma after trephination, lift the flap of the incised cornea at one end using fine-point surgical forceps under a bright-field microscope. Use an angled blunt spatula to dissect the stromal tissue along a lamellar plane on the posterior edge of the trephination. Continue the dissection process with careful, fine sweeping motions along the plane until the flap is completely detached from the cornea. Remove and discard the excised cornea.

### Data Analysis

After the keratectomies were performed, anterior segment OCT was used to image the eyes. The OCT images of the cornea were then analyzed by Image J to measure the depth of the cut. The depth of the cut was analyzed by measuring the thickness of the wounded cornea and the adjacent intact cornea. The percentage of excised cornea was calculated based on these two measurements by the following:

(thickness of intact cornea – thickness of wounded cornea) / thickness of intact cornea × 100.

### Statistical Analysis

Means, standard deviations, and *p*-values were calculated in Microsoft Excel 2016. Significance was determined using a two-tailed Student's *t*-test, and *p*-values < 0.05 were considered to be significant. Pearson's coefficient was calculated using the PEARSON function in Microsoft Excel 2016.

##  RESULTS

**Figure 1:**
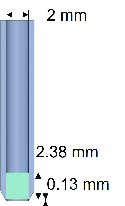
Schematic representation and illustration of the inner stopper guarded trephine.

Figure 1 illustrates the sagittal cross-section of the inner-stopper guarded trephine. The 2.38 mm measurement refers to the inner stopper. The 0.13 mm measurement represents the trephination depth calibrator that is removed during the assembly of the trephine.

**Figure 2:**
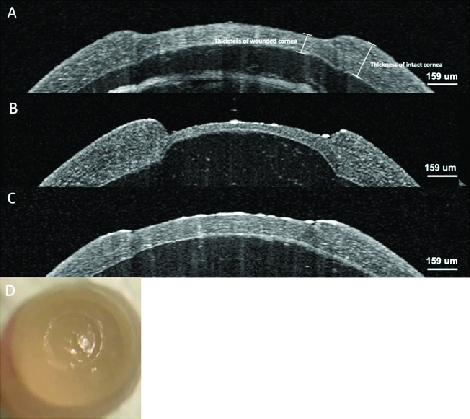
Optical coherence tomography (OCT) of the rat corneas after ALK. (A) Intermediate defect of the cornea. (B) Deep defect of the cornea. (C) Superficial defect of the cornea. (D) Rat eyeball after ALK with the inner-stopper guarded trephine.

**Figure 3:**
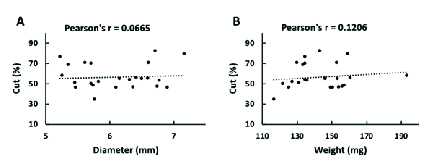
Correlations between the diameter (A) and weight (B) of the eyes and the percentage of cornea excised by the inner-stopper guarded trephine. The linear dependence was considered weak when the Pearson's *r* value is below 0.2.

**Figure 4:**
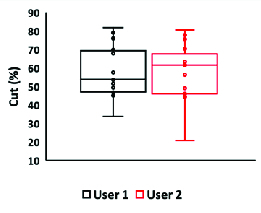
Comparison of corneal defects created by the inner-stopper guarded trephine by two users with different keratectomy experiences. User one had six months of experience in performing keratectomies and User two had one month of experience. There was no significant difference in the percentage of cornea excised between the two users.

**Figure 5:**
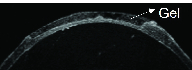
OCT image of a rat corneal defect filled in with a novel in situ forming therapeutic hydrogel.

OCT images in Figure 2 show the range of defect depths possible by this method, from shallow (approximately 20%) to deep (approximately 80%). Panel A shows an intermediate defect, whereas panel B shows a deeper defect and near-perforation of the cornea. Panel C shows a more superficial defect and panel D shows a photo of the *ex vivo *rat eyeball after introducing the defect.

The degree of defect, represented by the percentage of excised cornea, were graphed alongside the diameter and weight of the rat eyeballs. Figure 3 shows the linear relationship between these measurements and the defects produced by the inner-stopper guarded trephine. The weight (Pearson's *r* = 0.0665) and diameter (Pearson's *r* = 0.1206) of the rat eyes had a weak positive correlation with the percentage of excised cornea.

Figure 4 shows the consistency of the corneal defect depth between two different users. User two had about one month of keratectomy experience, compared to user one who had six months of keratectomy experience. On average, user one used the inner-stopper trephine to cut approximately 56.5% of the cornea with a standard deviation of 12.4%. Out of twenty-six eyes, two were perforated and discarded (7.7% perforation). The relative standard deviation for the percentage of corneal stromal tissue excised was 21.9%. The minimum percentage was found to be 33.8% and the maximum was 81.7%.

User two cut approximately 57.4% of the cornea with a standard deviation of 15.2%. The relative standard deviation for the percentage of cornea cut was 26.4%. The minimum defect was 20.9% and the maximum was 80.5%. Out of the 24 eyes, user two perforated 5 eyes. There was no significant difference between the values for weight (*p* = 0.7218), diameter (*p* = 0.6842), and percentage of cornea excised (*p* = 0.8569) between the two users, indicating that the procedure is consistent between the two users. The perpendicularity of the stromal defects were also objectively measured. On average, user one created defects that had an angle of 135.7°, with a standard deviation of 32.7. User two created defects that had an angle of 136.7°, with a standard deviation of 22.9. While this is less than ideal for the use of corneal transplantation and donor-recipient matching, this method nonetheless creates substantial stromal defects that can be filled with *in situ* forming hydrogels similar to what can be done in rabbits.^[8,9,10]^

Figure 5 shows an OCT image of the application of an *in situ* forming hydrogel to a corneal defect introduced by the described method.

##  DISCUSSION 

In many cases, keratectomies performed in rat models are irregular and vary significantly between different cornea samples. The protocol in this study describes a simple modification of a trephine that produces a relatively consistent corneal keratectomy wounds in rats.

When using the trephine, it is critical to ensure that the circular blade of the trephine is positioned over the center of the eye. If not centered, the size of the defect will be less accurate due to the changing corneal thickness that is dependent on the location where the trephine is placed on the cornea.^[11]^ Because rat corneas are considerably thinner than those of other larger animal models, there is a much decreased margin for error and greater risk of perforation. Another vital aspect of performing the keratectomy is to take advantage of the shear stress of the trephine blade by using extensive rotation and avoiding excess perpendicular pressure to the cornea, which can also result in perforation.

In the beginning stages of using this method, there may be difficulty in producing incisions in the cornea. One major complication is that the blade may glide along the surface of the cornea when one is trying to make an incision. To avoid this, the surgeon could attempt to gently pat the surface of the cornea with a tissue or sterile cotton swab. When performing the keratectomy, the eye should be immobilized. Any movement of the eyeball will reduce the amount of force imposed on the circular defect and potentially cause the trephine to glide along the surface of the cornea and scrape the surrounding layer of epithelial cells. For animal surgery, globe movement can be restricted by using ring forceps.

This simple trephine modification allows for the creation of uniform corneal defects between animals to study corneal wound healing in a very cost-effective way. Once the defects are created, researchers can attempt to treat the wound using various methods and analyze wound healing clinically and histopathologically without worrying about wound uniformity between surgeries and between surgeons. In rat eyes, the trephine seems to create oblique defects rather than perpendicular defects. We propose that this is due to the biomechanics, small size, and high curvature of the rat cornea. After the trephine is inserted to cut the stroma and removed, the elasticity of the now substantially thinned cornea may have caused some of the tissue to move back toward the center of the defect. Additionally, the high curvature of the rat cornea makes it more difficult for the trephine to cut a perpendicular angle in the stroma. Despite this difficulty to create perpendicular defects, this method is still useful to study therapies that can conform to the shape of the defect, such as hydrogels. We found in our prior published work that wound perpendicularity could be achieved reliably in rabbits, which have substantially thicker corneas with different biomechanical properties and lower curvature compared to those of rats.^[7]^ Compared to other methods such as mechanical keratectomy, excimer laser ablation, and microkeratomes, using an inner-stopper guarded trephine is more economical and simpler to use. For studies involving larger animal models, the protocol to create the inner-stopper trephine can be modified and replicated by using a larger biopsy punch to create a circular incision with a larger diameter.

In summary, we reported a simply designed inner-stopper guarded trephine-based protocol for producing consistent stromal defects in rat corneas, which is critical to study the effects of various therapeutics on corneal wound healing in a rat model. The inner stopper is essential for controlling the trephination depth.

##  Financial Support and Sponsorship

The authors would like to acknowledge the support from the National Institutes of Health (National Eye Institute K08EY028176 and a Departmental P30-EY026877 core grant), the Stanford SPARK Translational Research Grant and Maternal & Child Health Research Institute (MCHRI) (D.M.), the core grant and Career Development Award from Research to Prevent Blindness (RPB), the Matilda Ziegler Foundation, the VA Rehabilitation Research and Development Small Projects in Rehabilitation Effectiveness (Spire) program (I21 RX003179), and the Byers Eye Institute at Stanford.

##  Conflicts of Interest 

None.
